# A single nucleotide polymorphism associated with reduced alcohol intake in the *RASGRF2* gene predicts larger cortical volumes but faster longitudinal ventricular expansion in the elderly

**DOI:** 10.3389/fnagi.2013.00093

**Published:** 2013-12-19

**Authors:** Florence F. Roussotte, Boris A. Gutman, Derrek P. Hibar, Neda Jahanshad, Sarah K. Madsen, Clifford R. Jack, Michael W. Weiner, Paul M. Thompson, for the Alzheimer’s Disease Neuroimaging Initiative (ADNI)

**Affiliations:** ^1^Imaging Genetics Center, University of Southern CaliforniaLos Angeles, CA, USA; ^2^Departments of Neurology and Psychiatry, David Geffen School of Medicine at University of California Los AngelesLos Angeles, CA, USA; ^3^Mayo ClinicRochester, MN, USA; ^4^Departments of Radiology, Medicine, Psychiatry, University of California San FranciscoSan Francisco, CA, USA; ^5^Department of Veterans Affairs Medical CenterSan Francisco, CA, USA; ^6^Departments of Neurology, Psychiatry, Pediatrics, Engineering, Radiology, and Ophthalmology, Keck University of Southern California School of Medicine, University of Southern California, Los Angeles, CA, USA

**Keywords:** neuroimaging genetics, ventricular expansion, aging neuroscience, rasgrf2, brain volume, structural MRI

## Abstract

A recent genome-wide association meta-analysis showed a suggestive association between alcohol intake in humans and a common single nucleotide polymorphism in the ras-specific guanine nucleotide releasing factor 2 gene. Here, we tested whether this variant – associated with lower alcohol consumption – showed associations with brain structure and longitudinal ventricular expansion over time, across two independent elderly cohorts, totaling 1,032 subjects. We first examined a large sample of 738 elderly participants with neuroimaging and genetic data from the Alzheimer’s Disease Neuroimaging Initiative (ADNI1). Then, we assessed the generalizability of the findings by testing this polymorphism in a replication sample of 294 elderly subjects from a continuation of the first ADNI project (ADNI2) to minimize the risk of reporting false positive results. The minor allele – previously linked with lower alcohol intake – was associated with larger volumes in various cortical regions, notably the medial prefrontal cortex and cingulate gyrus in both cohorts. Intriguingly, the same allele also predicted faster ventricular expansion rates in the ADNI1 cohort at 1- and 2-year follow up. Despite a lack of alcohol consumption data in this study cohort, these findings, combined with earlier functional imaging investigations of the same gene, suggest the existence of reciprocal interactions between genes, brain, and drinking behavior.

## INTRODUCTION

A recent genome-wide association meta-analysis of alcohol intake in humans showed a suggestive association with a common variant (rs26907) in the *RASGRF2* gene (Schumann et al., 2011). Each copy of the minor A allele at rs26907 (MAF = 0.17), was associated with about 2.6% lower alcohol consumption (Schumann et al., 2011). Functional analysis of the *RASGRF2* gene by the same group revealed that alcohol preference is associated with whole-brain *RASGRF2* mRNA expression and that *RASGRF2* regulates alcohol-induced reinforcement by affecting mesolimbic neuron activity and dopamine release (Stacey et al., 2012).

Ras-specific guanine nucleotide-releasing factor 2 (Ras-GRF2) is a protein encoded by the *RASGRF2* gene. It is involved in signal transduction from ion channel receptors (Tian et al., 2004) and the regulation of MAPK signaling cascades, including the ERK pathway (Fasano and Brambilla, 2011; Feig, 2011), which plays a critical role in drug-induced reinforcement and synaptic plasticity (Girault et al., 2007). *RASGRF2* also helps to regulate neuronal excitability, neuronal survival in response to ischemia, learning and memory formation, and carcinogenesis (Jin and Feig, 2010; Feig, 2011; Fernandez-Medarde and Santos, 2011).

A growing enigma in neurology is how alcohol intake, and related genes, affect brain aging. *Moderate* alcohol intake protects against cardiovascular events (Rimm et al., 1999; Mukamal et al., 2006; Hvidtfeldt et al., 2010), but it is less clear how moderate drinking affect the brain over the long term. Elderly people who drink moderately may have fewer white matter abnormalities and infarcts than both non-drinkers and heavy drinkers (Mukamal et al., 2001; den Heijer et al., 2004; Mukamal, 2004). Genetic factors affect alcohol intake (Olfson and Bierut, 2012; Blaine et al., 2013; Meyers et al., 2013) but we do not know if the same genes affect brain structure and the rate of brain atrophy.

Dynamic changes in the brain’s lateral ventricles reveal the rate of brain atrophy as we age and reflect brain tissue loss with high effect sizes (Hua et al., 2013). Lateral ventricle expansion accompanies gray and white matter degeneration globally and in nearby subcortical regions (Ferrarini et al., 2008). The ventricles do not play a role in cognition, but ventricular expansion is associated with many brain-related health factors in the elderly, including current cognitive status and future memory decline (Coffey et al., 2001).

Here, we hypothesized that this candidate variant for reduced drinking (the minor A allele at the rs26907 locus in *RASGRF2*) might be associated with structural differences in fronto-limbic regions relevant to alcohol reinforcement in elderly subjects. To test if the findings generalized, we examined an independent replication sample of elderly participants, reducing the risk of false positive findings. We predicted that this variant might be associated with the ventricular expansion rate, in the larger sample whose ventricular volumes were measured at baseline, and at 1- and 2-year follow ups.

## MATERIALS AND METHODS

### SUBJECTS

Data used in preparing this article were obtained from the Alzheimer’s Disease Neuroimaging Initiative (ADNI) database^1^. ADNI, followed by ADNI-GO and ADNI-2, recruited over 1500 adults, ages 55–90, to participate in the research, consisting of cognitively normal older individuals, people with early or late mild cognitive impairment (MCI), and Alzheimer’s disease (AD). Subjects originally recruited for ADNI-1 and ADNI-GO had the option to be followed in ADNI-2. For up-to-date information, see .

Here, we analyzed two independent samples of elderly subjects with neuroimaging and genome-wide data from the ADNI1 and ADNI2 cohorts. We refer to ADNI-GO and ADNI-2 participants as “ADNI2,” as the only distinction was the grant funding that supported data collection, and the data collection was identical for them both. All ADNI studies are conducted according to the Good Clinical Practice guidelines, the Declaration of Helsinki, and U.S. 21 CFR Part 50 (Protection of Human Subjects), and Part 56 (Institutional Review Boards). Written informed consent was obtained from all participants before protocol-specific procedures were performed. To avoid the known effects of population stratification on genetic analysis (Lander and Schork, 1994), we only included non-Hispanic Caucasian subjects identified by self-report and confirmed by multi-dimensional scaling (MDS) analysis (Stein et al., 2010) in both ADNI cohorts.

#### Alzheimer’s disease neuroimaging initiative1

The ADNI1 cohort included three diagnostic groups: people with AD, MCI, and healthy elderly (cognitively normal) participants. We included participants from all diagnostic groups, as power is limited when performing any genetic association analysis. Typical effects of candidate genes on the phenotype are often around 1% of the mean value per allele (Stein et al., 2012), so we have often been able to pick up effects only when ADNI’s full sample is included. Even so, we have been able to replicate effects from ADNI in other non-overlapping samples, showing that affects found in ADNI can be robust and can generalize (Stein et al., 2011; Hibar et al., 2013; Roussotte et al., 2013). Effect sizes for individual genetic variants on brain structure in particular are expected to be small, so the genetic analysis would be underpowered if we further subdivided the sample (Stein et al., 2012). Our final analysis comprised 738 individuals (average age ± s.d. = 75.52 ± 6.78 years; 438 men/300 women) including 173 AD, 359 MCI, and 206 healthy participants (**Table 1**).

**Table 1:** Demographic and genetic data for the ADNI1 cohort.

ADNI1	Males	Females	Total
Total	438	300	738
Healthy elderly	112	94	206 (28%)
MCI	231	128	359 (49%)
AD	95	78	173 (23%)
rs26907 0 A alleles	318	226	544 (74%)
rs26907 1 A alleles	114	67	181 (24%)
rs26907 2 A alleles	6	7	13 (2%)
Mean age (±sd)	75.90 (±6.76)	74.98 (±6.78)	75.53 (±6.78)

#### Alzheimer’s disease neuroimaging initiative2

The ADNI2 cohort also included people with MCI who were further subdivided into early and late MCI (EMCI, LMCI). When we conducted these analyses, just under 300 ADNI2 subjects had been genotyped and processed using tensor based morphometry (TBM; see Minimal Deformation Target and Tensor Based Morphometry). Our final analysis comprised 294 individuals (average age ± s.d. = 73.16 ± 7.33 years; 166 men/128 women) including 25 AD, 66 LMCI, 81 EMCI, and 122 healthy participants (**Table 2**).

**Table 2:** Demographic and genetic data for the ADNI2 cohort.

ADNI2	Males	Females	Total
Total	166	128	294
Healthy elderly	64	58	122 (41%)
EMCI	47	34	81 (28%)
LMCI	38	28	66 (22%)
AD	17	8	25 (9%)
rs26907 0 A alleles	117	88	205 (70%)
rs26907 1 A alleles	46	38	84 (28%)
rs26907 2 A alleles	3	2	5 (2%)
Mean age (±sd)	74.49 (±7.14)	71.45 (±7.25)	73.16 (±7.33)

### GENOTYPING AND SNP SELECTION

In ADNI, genome-wide association study (GWAS) data was collected from 1252 participants. All 818 subjects (including the non-Caucasians not used in this study) from the ADNI1 sample were genotyped using the Illumina Human 610-Quad BeadChip (San Diego, CA, USA), and DNA samples were genotyped from 434 ADNI-GO/ADNI-2 participants using the Illumina OmniExpress genotyping array.

Data from both cohorts were imputed to a common reference space; the 1000 genomes CEU (Caucasian) reference set following freely available imputation protocols (ENIGMA2, 2012). The imputed data were filtered for standard imputation quality criteria (imputation quality: Rsq < 0.3) and minor allele frequency (MAF < 0.05). The final, filtered genetic datasets were used for our genetic analyses.

We analyzed a common (G/A, minor allele frequency: *A* = 0.170) single nucleotide polymorphism (rs26907) in the ras-specific guanine nucleotide releasing factor 2 (*RASGRF2*) gene previously implicated in alcohol consumption (Schumann et al., 2011; Stacey et al., 2012) for association with regional brain volumes in both ADNI cohorts, and longitudinal ventricular expansion in the ADNI1 subjects who had been included in the regional brain volumes association study. The allele distribution in both samples did not deviate from the Hardy-Weinberg equilibrium (*p* = 0.6118 for ADNI1 and *p* = 0.1912 for ADNI2).

### IMAGE ACQUISITION

ADNI1 subjects were scanned with a standardized MRI protocol developed for this cohort (Leow et al., 2006; Jack et al., 2008). Briefly, high-resolution structural brain MRI scans were acquired at 58 sites across North America, using 1.5 T MRI scanners. A sagittal 3D MP-RAGE sequence was used, optimized for consistency across sites (Jack et al., 2008; TR/TE = 2400/1000 ms; flip angle = 8°; FOV = 24 cm; final reconstructed voxel resolution = 0.9375 × 0.9375 × 1.2 mm^3^). Each ADNI2 subject received a 3 T accelerated T1-weighted MRI scan. By vendor, General Electric (GE) scanners use IR-SPGR sequences and Philips and Siemens use MP-RAGE sequences. Scan vendors and sequences for ADNI2 are available online.

### REGIONAL BRAIN VOLUMES ASSOCIATION STUDIES (ADNI1 AND ADNI2)

#### Image correction and pre-processing

For both ADNI samples, image corrections were applied using a processing pipeline at the Mayo Clinic, consisting of: (1) a procedure termed GradWarp to correct geometric distortion due to gradient non-linearity (Jovicich et al., 2006), (2) a “B1-correction”, to adjust for image intensity inhomogeneity due to B1 non-uniformity using calibration scans (Jack et al., 2008), (3) “N3” bias field correction, for reducing residual intensity inhomogeneity (Sled et al., 1998), and (4) geometrical scaling, according to a phantom scan acquired for each subject (Jack et al., 2008) to adjust for scanner- and session-specific calibration errors^2^. To adjust for global differences in brain positioning and scale, all subjects’ scans were linearly registered to the stereotaxic space defined by the International Consortium for Brain Mapping (ICBM-53; Mazziotta et al., 2001), using a 9-parameter (9P) transformation (three translations, three rotations, three scales (Collins et al., 1994). For both ADNI cohorts, we used standard trilinear interpolation and resampled the resulting aligned scans to have 1mm isotropic voxels. Subjects’ brain images were not skull-stripped during pre-processing.

#### Minimal deformation target and tensor based morphometry

For ADNI1, we created a minimal deformation target (MDT), which serves as an unbiased average template image for automated image registration, and to reduce statistical bias. The MDT was created using the MRI scans of 40 randomly selected healthy elderly subjects, as detailed elsewhere (Hua et al., 2008a,b). The MDT image was calculated as a geometrically centered mean anatomical image, using a method called sKL-MI to align data to an average affine registered target image; this procedure leads to fairly “sharp” average brain image for a group and follows a procedure we developed and tested elsewhere (Hua et al., 2008a,b).

To quantify 3D patterns of volumetric tissue variations, all individual T1-weighted images (*N* = 1,032) were non-linearly aligned to the MDT template created for ADNI1 with an inverse-consistent 3D elastic warping technique using a mutual information cost function (Leow et al., 2005). For each subject, a separate Jacobian matrix field was derived from the gradients of the deformation field that aligned that individual brain to the MDT template. The determinant of the local Jacobian matrix was derived from the forward deformation field to characterize local volume differences. Color-coded Jacobian determinants were used to illustrate regions of volume expansion, i.e., those with det J(r) >1, or contraction, i.e., det J(r) <1 (Freeborough and Fox, 1998; Thompson et al., 2000; Chung et al., 2001; Riddle et al., 2004) relative to the template. All images were registered to the same template, so these Jacobian maps shared common anatomical coordinates, defined by the normal template. Individual Jacobian maps were retained for further statistical analyses.

#### Regression of structural brain differences with the candidate SNP

In both ADNI cohorts, we investigated how the rs26907 variant affected regional brain volumes using univariate linear regression to associate the number of minor A alleles (0,1, or 2) with the Jacobian values (describing the amount of brain tissue deficit or excess relative to the standard template) at each voxel in the brain, after covarying for age, sex, and diagnosis (i.e., AD, MCI, and healthy elderly for ADNI1 and AD, LMCI, EMCI, and healthy elderly for ADNI2).

#### Multiple comparisons correction

Computing thousands of association tests across the brain can introduce a high Type I (false positive) error rate in neuroimaging studies, if not controlled. We used a searchlight method for false discovery rate (FDR) correction (Langers et al., 2007), which controls the FDR in any reported statistical map. This corrected the map of statistical associations between the image phenotype (morphometry) and genotype at the rs26907 locus. All maps shown are thresholded at the appropriate corrected *p*-value, after performing searchlight FDR (*q* = 0.05), to show only regions of significance that passed the multiple comparisons correction.

### LONGITUDINAL VENTRICULAR EXPANSION STUDY (ADNI1)

#### Image correction and pre-processing

For lateral ventricle segmentation, we analyzed baseline (*N* = 738), 1-year (*N* = 623), and 2-year (*N* = 481) follow-up brain MRI scans from all the ADNI1 subjects included in the regional brain volumes association study described above. Raw MRI scans were pre-processed to reduce signal inhomogeneity and linearly registered to a template (using 9 parameter registration). Segmentations were assessed visually for defects from multiple views. All subjects were quality controlled for ventricular segmentation, and one baseline ADNI1 subject was removed from the longitudinal ventricular expansion study after quality control of the ventricular surfaces. Our final analysis thus included 737 ADNI1 subjects at baseline, 623 at 12-month follow-up, and 481 at 24-month follow-up.

#### Segmentation of the lateral ventricles

Prior methods for ventricular segmentation have used semi-automated, automated (Chou et al., 2008), and single-atlas or multi-atlas methods (Chou et al., 2009). Here we segmented the ventricles with our modified multi-atlas approach (Gutman et al., 2013; Madsen et al., 2013). Our segmentation approach uses group-wise surface registration to existing templates in addition to surface-based template blending to yield more accurate results. The lateral ventricles were segmented in each subject using a validated method (Chou et al., 2008). Ventricular surfaces were then extracted from these segmentations and an inverse-consistent fluid registration with a mutual information fidelity term aligned a set of hand-labeled ventricular templates to each scan (Leow et al., 2007). The template surfaces were registered into homologous point-to-point correspondence as a group using medial-spherical registration (Gutman et al., 2012).

To construct a surface boundary of the new subject, a normalized similarity measure between each template image and the new image was computed in a neighborhood around each vertex point of each deformed template surface. The position of each point of the new boundary was defined by the template, which showed the best similarity score, here normalized mutual information. The final surface was then constrained to be a smooth approximation of this winner-takes-all construction. This approach is very similar to that of (Yushkevich et al., 2010), except ours is based on surface geometry rather than voxels in an image. The approach is advantageous compared to whole-template approaches typically used in multi-atlas segmentation, allowing more flexible segmentation, particularly for outliers.

#### Statistical analyses: associations of rs26907 genotype with ventricular volumes

Statistical tests using the number of minor A alleles at the rs26907 locus to predict left and right ventricular volumes were conducted with SPSS 21.0. This assumes an additive model of allele effects – a common assumption. We tested general linear models (GLMs) with outcome variables of ventricular volume at baseline [*N* = 737, one subject was excluded as mentioned in Section “Image Correction and Pre-Processing” under Longitudinal Ventricular Expansion Study (ADNI1)], difference between ventricular volume at baseline and volume after 1 year (in cubic mm, *N* = 623), difference between ventricular volume at baseline and volume after 2 years (in cubic mm, *N* = 481), and covarying for age, sex, and diagnosis (i.e., healthy elderly control, MCI, or AD). As the volume images were already normalized for overall brain size during the 9-parameter affine alignment, additional volume normalization was unnecessary. It would only have obscured the effect of faster relative expansion rates. As our expansion rates are computed over some snapshot in time - here only 1 or 2 years – the variation in rate of expansion, after being normalized for overall brain size, would have already had an effect on total volume prior to baseline image acquisition. In all analyses, the left and right ventricular volumes were tested separately and combined.

## RESULTS

### REGIONAL BRAIN VOLUMES ASSOCIATION STUDIES

**FIGURE 1:**
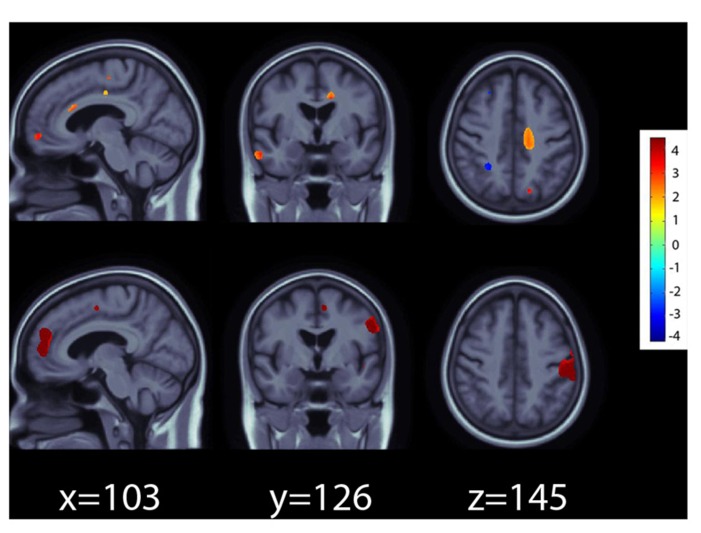
**Effects of the A allele at the rs26907 locus in the *RASGRF2* gene, on regional brain volumes in the ADNI1 (top panel) and ADNI2 (bottom panel) cohorts**. Positive beta values (*warm colors*) show regions where the minor A allele was associated with greater tissue volumes. Negative beta values *(cool colors*) show regions where the minor A allele was associated with lower tissue volumes. The color bar encodes the average percentage of volume difference relative to the template associated with the minor allele. Tests for associations are adjusted for age, sex, and diagnosis; maps are corrected for multiple comparisons with the searchlight false discovery rate (FDR) method at *q* = 0.05. Images are in radiological convention (left side of the brain shown on the right).

In the ADNI1 sample, the *RASGRF2* polymorphism rs26907 predicted differences in regional brain volumes, after covarying for sex, age, and diagnosis, and after multiple comparisons correction at *q* = 0.05 (**Figure 1**, top panel). Larger volumes in the medial prefrontal cortices, cingulate gyrus, and right temporal lobe, were statistically related to carrying the minor A allele at the rs26907 locus. Regional volume differences associated with the minor allele ranged from 2 to 4%.

The *RASGRF2* polymorphism rs26907 also predicted differences in regional brain volumes in the ADNI2 cohort, after covarying for sex, age, and diagnosis, and after multiple comparisons correction at *q* = 0.05 (**Figure 1**, bottom panel). As in ADNI1, the minor A allele was associated with larger volumes in the medial prefrontal cortices and cingulate gyrus. It also predicted larger volumes in the postcentral gyrus. Regional volume differences associated with the minor allele ranged from 3 to 5%.

In the ADNI1 cohort, the minor A allele was also associated with smaller volumes in the cerebellum, but this was detectable in the ADNI2 cohort only when a less conservative threshold for multiple comparisons correction at *q* = 0.10 was used (data not shown) possibly because the ADNI2 sample was smaller and afforded less statistical power to detect small gene effects on the brain.

### LONGITUDINAL VENTRICULAR EXPANSION STUDY

**FIGURE 2:**
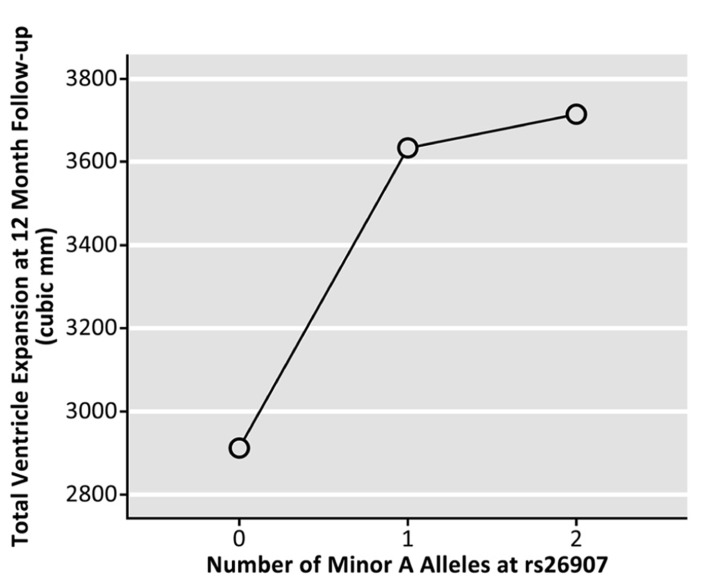
**Effects of the A allele at the rs26907 locus in the *RASGRF2* gene, on total longitudinal ventricular expansion in the ADNI1 cohort at 12-month follow-up (*N* = 623)**.

**FIGURE 3:**
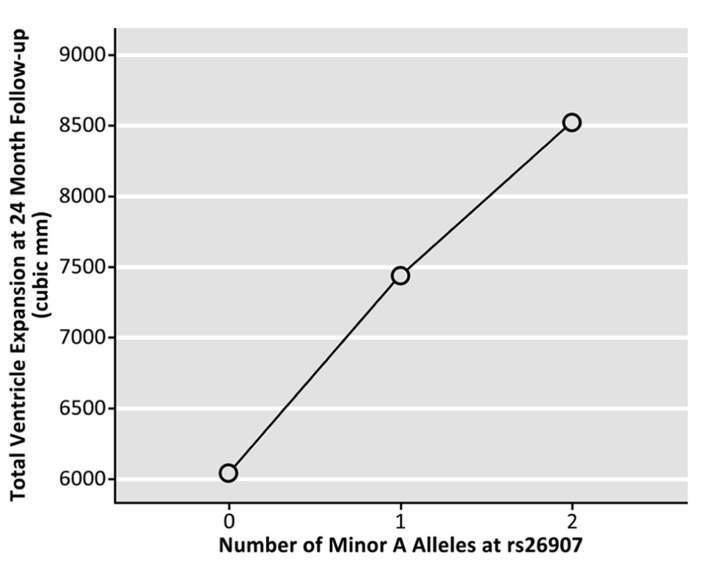
**Effects of the A allele at the rs26907 locus in the *RASGRF2* gene, on total longitudinal ventricular expansion in the ADNI1 cohort at 24-month follow-up (*N* = 481)**.

Genotype at the rs26907 locus was not significantly related to baseline volumes of the left (*p* = 0.173) or right (*p* = 0.629) lateral ventricles, after sex, age, and diagnosis were regressed out (*N* = 737). To determine if the allele was related to rates of brain tissue loss, we then examined the differences between ventricular volumes at baseline and volumes after 1 year (in cubic mm, *N* = 623), and 2 years (in cubic mm, *N* = 481), after covarying for sex, age, and diagnosis. Ventricular expansion rates relative to overall brain size showed a significant correlation with the number of A alleles at rs26907. After 1 year, carrying more minor A alleles was associated with greater rates of expansion in the left (*p* = 0.040) and right ventricle (*p* = 0.010), and with a greater overall rate of ventricular expansion (*p* = 0.017, **Figure 2**; *N* = 623). A similar pattern was observed at 2-year follow-up. Carrying more minor A alleles at the rs26907 locus was associated with faster ventricle expansion in the left (*p* = 0.030) and right ventricle (*p* = 0.028), and with greater total ventricle expansion (*p* = 0.024, **Figure 3**; *N* = 481).

## DISCUSSION

This study is the first to report an association between brain structure, longitudinal ventricular expansion, and a common single nucleotide polymorphism (rs26907) associated with alcohol intake (Schumann et al., 2011; Stacey et al., 2012) in the gene encoding ras-specific guanine nucleotide releasing factor 2. The variant previously associated with lower alcohol consumption predicted larger cortical volumes in the elderly at baseline. We replicated these findings in an independent elderly cohort despite a smaller sample size with lower statistical power, suggesting that the association of this variant with larger cortical volumes in the elderly may be independent of age, sex, or disease status. The same allele associated with reduced alcohol intake also predicted faster longitudinal ventricular expansion at 1- and 2-year follow up.

The rs26907 polymorphism in *RASGRF2* is associated with alcohol intake (Schumann et al., 2011; Stacey et al., 2012), and this study further links it to regional brain volumes, in several systems involved in alcohol reward – where cellular and molecular processes including dopamine release are also affected by this gene. It may seem surprising that the rs26907 allele associated with reduced alcohol intake – which is the less common one – predicts larger regional cortical volumes in the elderly at baseline but faster ventricular expansion rates over time, reflecting an accumulation of brain tissue loss globally, throughout multiple brain regions. In fact, baseline regional cortical volumes are likely to depend on factors unrelated to neurodegeneration, such as neurodevelopmental differences. Even so, if the lateral ventricles expand approximately linearly with age in healthy elderly individuals (Blatter et al., 1995), accelerated rates of ventricular expansion may be indicators or predictors of degenerative brain disorders (Apostolova et al., 2012).

The rs26907 polymorphism in *RASGRF2* was associated with regional brain volumes and longitudinal ventricular expansion, but we are unable to provide mechanistic evidence for how this variant might affect brain structure. Rs26907 is an intronic SNP that is not linked with any known changes in gene function. SNPs in non-coding regions can affect gene splicing, and *RASGRF2* may have one alternative splice variant (Feig, 2011). This polymorphism may affect transcription factor binding, mRNA degradation, and other molecular genetic processes that may relate to brain volumes. Data from the encyclopedia of DNA elements (“ENCODE”)^3^ as presented in HaploReg^4^, suggest that rs26907 alters the Rad21 regulatory motif. Since Rad21 is a transcription factor involved in apoptosis (Pati et al., 2002), programmed cell death represents a plausible mechanism by which this variant could affect brain volumes and rates of ventricular expansion, which indicate neuronal loss. Although we do not know the precise mechanisms that relate this polymorphism to brain structure, this particular variant may drive the observed associations, as there are no SNPs in high linkage disequilibrium (as defined by *r*^2^ > 0.8) with rs26907 in the 1000 Genomes Caucasian (CEU) panel^5^.

An important limitation of this study is that no alcohol consumption measures were available in these samples (although the cohorts excluded alcohol abusers). We cannot establish a relationship between the rs26907 variant and alcohol intake, or among brain volumes, longitudinal ventricular expansion, and alcohol intake in these particular cohorts. Some of these associations have been reported in other cohorts. Our experimental design does reveal whether the variant of interest directly affects the brain or just modifies drinking behaviors that affect brain structure and the rate of brain atrophy. This would be an interesting target of study. Nonetheless, the functional imaging literature may shed light on our results. A haplotype containing rs26907 as well as other SNPs in *RASGRF2* was associated with ventral striatal activity during reward anticipation in healthy 14-year old boys, suggesting a hypersensitive reward system (Stacey et al., 2012). Follow-up at age 16 showed an association between this haplotype and number of drinking episodes (Stacey et al., 2012). The existence of differences, first in brain activation and later in drinking behaviors, between young carriers of risk alleles in *RASGRF2* and carriers of protective alleles, suggests that this gene may directly affect the developing brain. This may result in different drinking patterns later in life. Along with other behaviors, this may further affect brain structure and function throughout the lifespan.

*RASGRF2* regulates alcohol-induced reinforcement and alcohol preference is associated with whole-brain *RASGRF2* mRNA expression (Stacey et al., 2012). During neurodevelopment, carriers of the rs26907 allele associated with reduced drinking may have developed neurological circuits conferring less sensitivity to alcohol reward. This could be one factor associated with their volume differences in the medial prefrontal cortex, which plays a role in the mesolimbic reward system (Baltazar et al., 2013). As a result, these individuals may have been more likely to abstain from drinking throughout their lives. In our study, this may be linked to the faster lateral ventricle expansion rates (typically associated with neurodegeneration across the brain) in elderly carriers of the allele. Elderly individuals who abstain from drinking have higher rates of brain abnormalities and infarcts than moderate drinkers (Mukamal et al., 2001; den Heijer et al., 2004; Mukamal, 2004). This framework remains speculative, and future studies relating alcohol consumption data to brain imaging and genetic data, should clarify the direction of these relationships. Even so, our findings, combined with earlier functional imaging investigations of the same gene (Stacey et al., 2012) point to interdependent and reciprocal interactions between genes, brain, and drinking behaviors.

## Conflict of Interest Statement

The authors declare that the research was conducted in the absence of any commercial or financial relationships that could be construed as a potential conflict of interest.

## AUTHOR CONTRIBUTIONS

Florence F. Roussotte formulated the hypothesis, conducted the analyses, and wrote the manuscript. Boris A. Gutman developed the novel Linear Discriminant Analysis (LDA)-based approach for segmenting the lateral ventricles, described in Section Longitudinal Ventricular Expansion Study. Derrek P. Hibar helped with data analysis and interpretation, and helped revise the manuscript. Neda Jahanshad helped with data analysis and interpretation, and helped revise the manuscript. Sarah K. Madsen helped with data analysis and interpretation, and helped revise the manuscript. Clifford R. Jack helped revise the manuscript. Michael W. Weiner is the principal investigator of the ADNI initiative and helped revise the manuscript. Paul M. Thompson is the principal investigator of this study, and overviewed all phases of the investigation and manuscript preparation.

## Footnotes

2

3

4

5

6
